# Severe Arrhythmia Complicating Takotsubo Cardiomyopathy Revealing an Underlying Pheochromocytoma: A Case Report

**DOI:** 10.7759/cureus.109885

**Published:** 2026-05-29

**Authors:** Meryem Tabat, Zineb El Jaouhari, Abdnacer Drighil

**Affiliations:** 1 Cardiology, Centre Hospitalier Universitaire (CHU) Ibn Rochd, Casablanca, MAR; 2 Cardiology, Ibn Rochd University Hospital, Casablanca, MAR

**Keywords:** cardiac arrythmia, catecholamine, catecholamine hypersecretion, electric shock, pheochromocytoma, takotsubo cardiomyopathy (ttc)

## Abstract

Pheochromocytoma is a rare catecholamine-secreting tumor that can cause major cardiovascular complications, including acute heart failure, arrhythmias, and stress-induced cardiomyopathy. Takotsubo syndrome (TTS) is an acute non-ischemic cardiomyopathy characterized by transient left ventricular systolic dysfunction without obstructive coronary artery disease, often mimicking acute coronary syndrome. Catecholamine excess plays a central role in its pathophysiology, leading to myocardial stunning, hemodynamic instability, and arrhythmias. The association between pheochromocytoma and TTS is uncommon and frequently associated with severe clinical presentations.

We report the case of a 52-year-old woman with poorly controlled hypertension treated with a calcium channel blocker, who presented with chest pain, dyspnea, and palpitations. On admission, blood pressure was 178/122 mmHg, and heart rate was 102 bpm. The patient described recurrent episodes of headache and palpitations occurring two to three times monthly and lasting 30 to 60 minutes, suggesting secondary hypertension.

Electrocardiography showed 1 mm ST-segment elevation in the anterior leads without reciprocal changes and a prolonged QTc interval (480 ms). Peak high-sensitivity troponin reached 1880 ng/L (reference <35 ng/L), while brain natriuretic peptide (BNP) was 450 pg/mL (reference <125 pg/mL). Transthoracic echocardiography revealed a left ventricular ejection fraction of 45% with apical akinesia and basal hyperkinesia, consistent with Takotsubo syndrome. Left ventriculography confirmed typical apical ballooning, and coronary angiography showed no obstructive coronary artery disease.

During hospitalization, the patient developed rapid atrial fibrillation with hemodynamic instability, requiring electrical cardioversion and amiodarone therapy. Labile blood pressure and paroxysmal symptoms raised suspicion of catecholamine excess, subsequently confirmed by elevated plasma and urinary metanephrines. Abdominal CT identified a 30 × 25 × 28 mm left adrenal mass with attenuation >10 HU and intense heterogeneous contrast enhancement, highly suggestive of pheochromocytoma. Preoperative treatment with phenoxybenzamine was progressively titrated to achieve hemodynamic stabilization before surgical resection.

After surgery, left ventricular systolic function completely recovered, with ejection fraction improving to 58%. At the sixth-week follow-up, plasma and urinary metanephrines had normalized, confirming complete biochemical remission and successful tumor removal. Blood pressure also normalized postoperatively. This case highlights the importance of considering pheochromocytoma in patients with Takotsubo syndrome complicated by severe arrhythmias or labile hypertension, as early diagnosis may improve management and prognosis.

## Introduction

Pheochromocytoma is a rare catecholamine-secreting tumor arising from adrenal chromaffin cells, associated with significant cardiovascular morbidity due to excessive adrenergic stimulation. Clinical manifestations range from paroxysmal hypertension to acute heart failure, myocardial injury, and malignant arrhythmias, driven by catecholamine-induced myocardial toxicity, vasoconstriction, and electrical instability [1].

Takotsubo syndrome (TTS), also known as Takotsubo cardiomyopathy (TTC), is an acute non-ischemic cardiomyopathy characterized by transient, reversible left ventricular systolic dysfunction in the absence of obstructive coronary artery disease, frequently mimicking an acute coronary syndrome. Although commonly triggered by emotional or physical stress, current pathophysiological concepts emphasize sympathetic hyperactivation and catecholamine excess as central mechanisms leading to myocardial stunning [2]. Experimental models further suggest that elevated epinephrine levels may induce a β2-adrenoceptor Gs-to-Gi signaling switch, contributing to severe but reversible myocardial dysfunction.

In some patients, this catecholamine-mediated myocardial injury may result in significant hemodynamic compromise and arrhythmic complications. In rare cases, pheochromocytoma may underlie TTC, resulting in more severe and atypical presentations with increased risk of cardiogenic shock and life-threatening arrhythmias. Recognizing this association is essential, as it has important diagnostic and therapeutic implications [3].

## Case presentation

The patient was a 52-year-old woman with no significant cardiovascular history apart from poorly controlled hypertension treated with a calcium channel blocker, who presented with chest pain, dyspnea, and palpitations. On detailed interrogation, the patient did not report any recent emotional or physical stress. However, she described recurrent paroxysmal episodes of headache and palpitations in the context of poorly controlled hypertension, raising suspicion of a secondary cause of hypertension.

**Figure 1:**
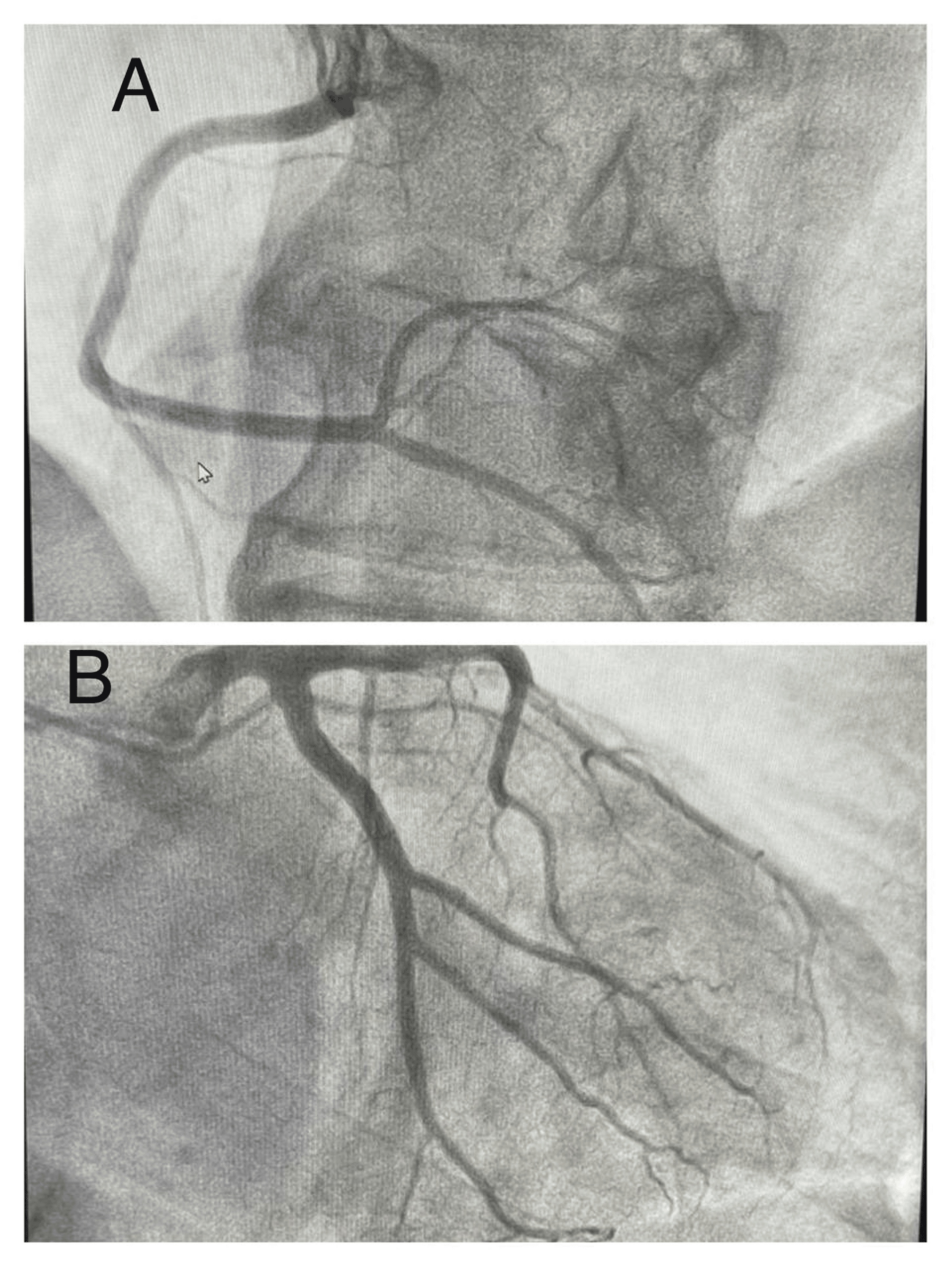
Coronary angiography performed via the right radial artery A: Right coronary artery angiographically normal; B: Left coronary artery without significant lesions

**Figure 2:**
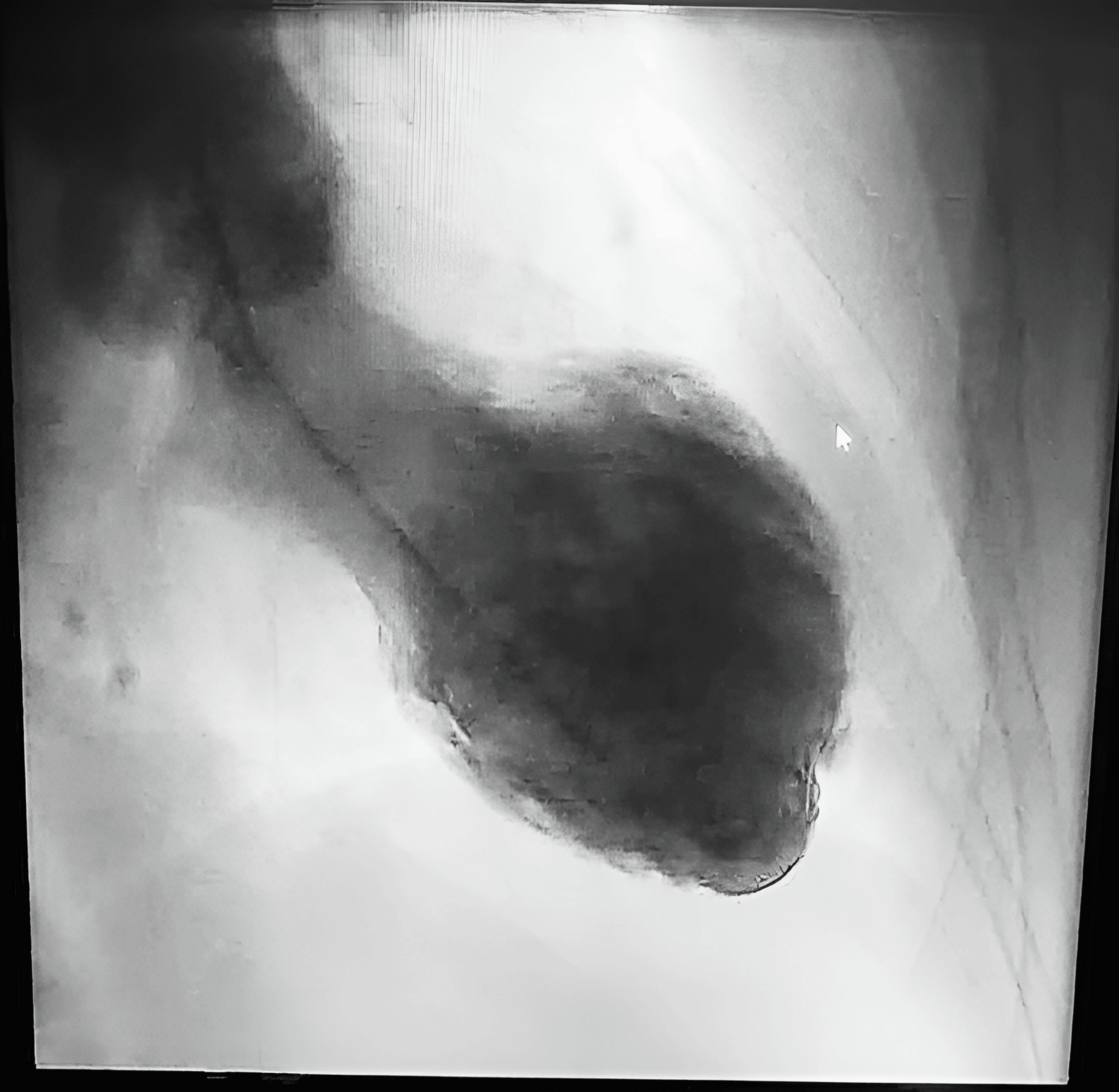
Ventriculography performed using a pigtail probe with right anterior oblique incidence reveals apical ballooning

Initial investigations showed sinus rhythm with 1 mm ST-segment elevation in leads V2-V3 without reciprocal changes, along with elevated cardiac biomarkers. Transthoracic echocardiography revealed findings consistent with TTC, with a left ventricular ejection fraction of 45%. Coronary angiography showed no evidence of obstructive coronary artery disease (Figure 1), and left ventriculography demonstrated apical ballooning (Figure 2).

**Figure 3:**
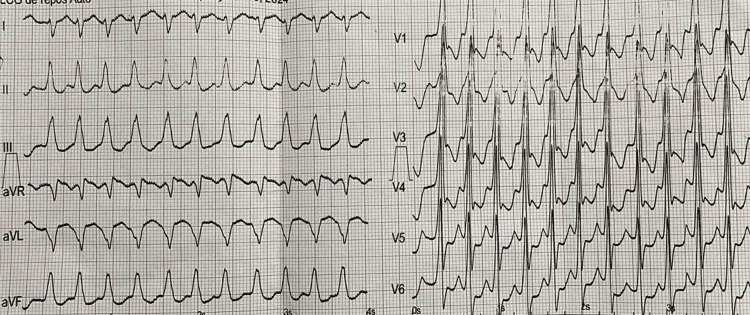
ECG showing a severe arrhythmia with a rapid ventricular response

During hospitalization, the patient developed a severe arrhythmia with rapid ventricular response (Figure 3), specifically rapid atrial fibrillation with a ventricular rate >150 bpm, leading to acute hemodynamic instability and a nadir blood pressure of 80/50 mmHg. Given the severity of hypotension and clinical deterioration, immediate electrical cardioversion was performed as first-line rhythm control rather than pharmacological rate control. This was followed by antiarrhythmic therapy with amiodarone (loading dose of 300 mg followed by 900 mg over 24 hours) and admission to the intensive care unit for close monitoring.

**Figure 4:**
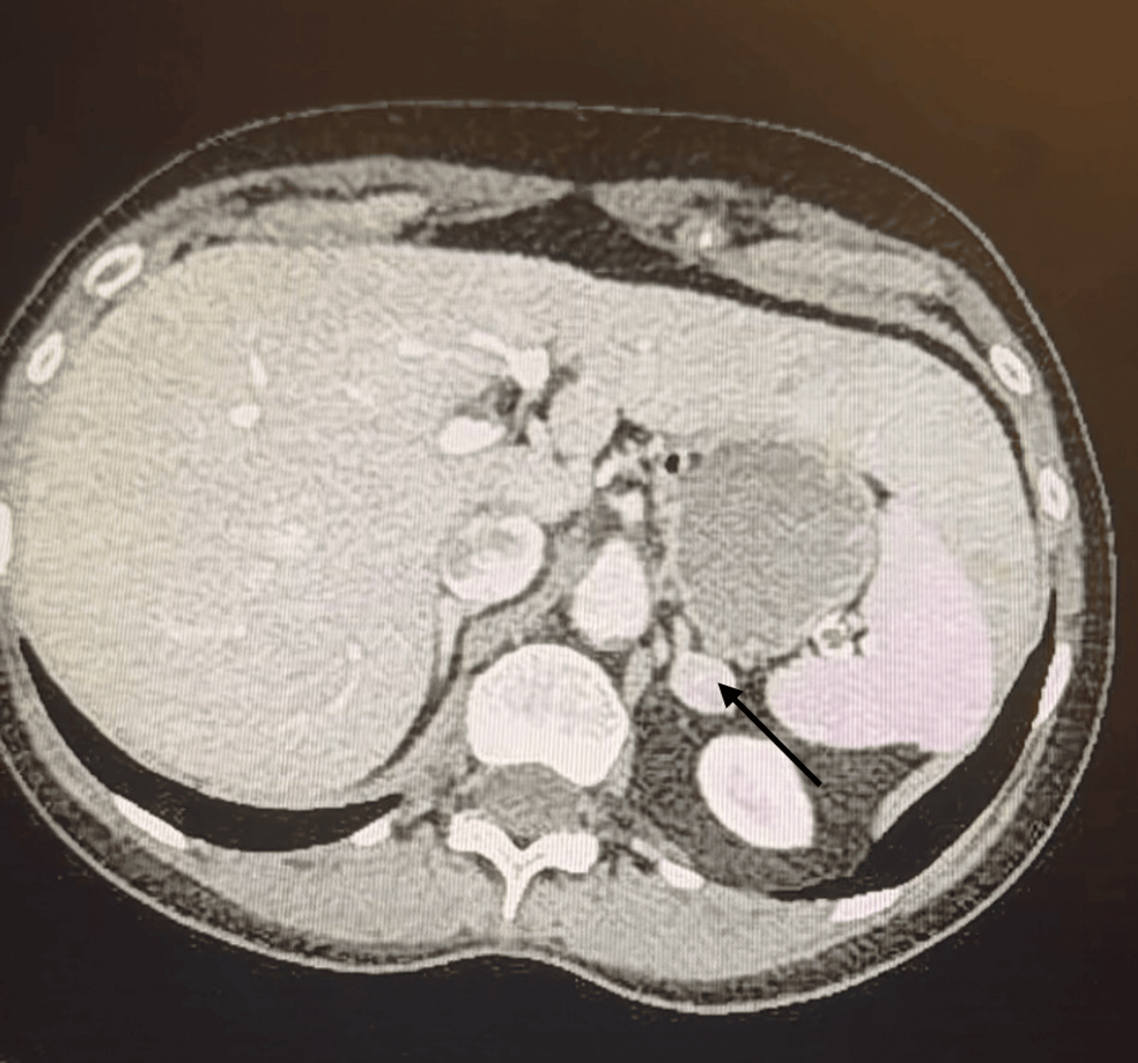
Abdominal CT revealing the adrenal mass (black arrow)

The occurrence of electrical instability associated with labile blood pressure, together with the history of paroxysmal headaches and palpitations in the absence of an identifiable stress trigger, strongly suggested an underlying catecholamine excess, particularly pheochromocytoma. The biochemical evaluation revealed markedly elevated plasma and urinary metanephrines, with plasma metanephrine levels of 4,850 pg/mL (normal <90 pg/mL) and normetanephrine levels of 6,200 pg/mL (normal <180 pg/mL). The 24-hour urinary fractionated metanephrines were also significantly increased at 3,800 µg/24 h (normal <600 µg/24 h), consistent with severe catecholamine excess and highly suggestive of pheochromocytoma. Abdominal CT imaging identified a left adrenal mass consistent with pheochromocytoma (Figure 4).

The patient was treated with appropriate alpha-adrenergic blockade followed by surgical resection of the adrenal tumor. Postoperatively, there was complete recovery of cardiac function and normalization of blood pressure. This case highlights that TTC complicated by severe arrhythmias should prompt investigation for secondary causes, particularly pheochromocytoma, as early diagnosis significantly impacts management and prognosis.

## Discussion

Takotsubo cardiomyopathy (TTC) is a transient left ventricular dysfunction most commonly triggered by acute stress, but it may also result from endogenous catecholamine excess, particularly in the setting of pheochromocytoma. This association represents a secondary form of TTC, often characterized by more severe clinical presentations [4,5]. The underlying pathophysiology is primarily related to catecholamine-induced myocardial toxicity, involving microvascular dysfunction, intracellular calcium overload, and oxidative stress, leading to myocardial stunning. Regional differences in β-adrenergic receptor density may explain the typical apical ballooning pattern [4,5].

Pheochromocytoma-associated TTC is associated with a higher rate of complications compared to primary TTC, including cardiogenic shock and severe arrhythmias [6,7]. Arrhythmias result from adrenergic overstimulation, promoting increased automaticity, triggered activity, and dispersion of repolarization [8,9]. While atrial fibrillation is common, ventricular arrhythmias and sudden cardiac death have also been reported. In the absence of an identifiable stress trigger, the presence of paroxysmal symptoms such as headache and palpitations, together with poorly controlled hypertension, should raise suspicion for pheochromocytoma in patients presenting with TTC. Diagnosis relies on elevated plasma or urinary metanephrines followed by imaging to localize the adrenal tumor [10].

Management requires initial alpha-adrenergic blockade before surgery, followed by tumor resection, which is usually associated with complete recovery of cardiac function [5,7]. This case emphasizes the importance of systematically considering pheochromocytoma in atypical or complicated forms of TTC, particularly when associated with severe arrhythmias.

## Conclusions

Takotsubo syndrome secondary to pheochromocytoma is a high-risk catecholamine-induced cardiomyopathy, with a poorer prognosis than primary stress-induced forms. It is characterized by persistent sympathetic hyperactivation leading to marked electrical instability and severe hemodynamic failure. The occurrence of atrial fibrillation may be considered a major prognostic warning sign, associated with an increased risk of cardiogenic shock and malignant ventricular arrhythmias. Early biochemical screening could enable prompt diagnosis and risk stratification. The early initiation of alpha-blocker therapy may help stabilize hemodynamic and electrical disturbances and improve prognosis.
